# Loss of miR-637 promotes cancer cell stemness via WASH/IL-8 pathway and serves as a novel prognostic marker in esophageal squamous cell carcinoma

**DOI:** 10.1186/s40364-022-00424-x

**Published:** 2022-11-03

**Authors:** Mengxing Guo, Jingyao Lian, Yaqing Liu, Bo Dong, Qianyi He, Qitai Zhao, Hongyan Zhang, Yu Qi, Yi Zhang, Lan Huang

**Affiliations:** 1 Biotherapy Center, The First Affiliated Hospital of Zhengzhou University, Zhengzhou, China; 2 State Key Laboratory of Esophageal Cancer Prevention & Treatment, Zhengzhou, China; 3 Department of Thoracic Surgery, The First Affiliated Hospital of Zhengzhou University, Zhengzhou, China

**Keywords:** Cancer stem cells, Esophageal squamous cell carcinoma, Interleukin-8, miR-637, WASH

## Abstract

**Background:**

Esophageal carcinoma is the highly lethal cancer in the world, predominantly in some areas of East Asia. We previously reported that overexpression of cytoskeleton regulator Wiskott-Aldrich syndrome protein and SCAR Homolog (WASH) associates with poor prognosis of patients with esophageal squamous cell carcinoma (ESCC). However, the molecular mechanism and clinical significance involved in WASH overexpression have not been fully elucidated.

**Methods:**

Bioinformatics analysis and luciferase reporter assay were used to predict and validate miR-637 as a regulator of WASH in ESCC cell lines. qRT-PCR, Western blotting and ELISA assays were performed to examine RNA expression and protein levels, respectively. Next, the biological functions of miR-637 were explored by tumor sphere formation assay in vitro and nude mouse tumor xenograft in vivo. Finally, we evaluated the association of miR-637 levels with clinical features in ESCC patients.

**Results:**

We identified miR-637 as a WASH-targeting miRNA. miR-637 mimic strongly attenuated the downstream IL-8 production and tumor sphere formation in esophageal cancer cells, whereas miR-637 inhibitor displayed an opposite effect. IL-8 could facilitate stem-like properties and partially rescue the phenotypes induced by miR-637 mimic. Furthermore, miR-637 inhibitor dramatically promoted IL-8 expression and cancer stemness properties in a WASH-dependent manner. Ectopic expression of miR-637 also inhibited tumor growth in a mouse model. Clinically, low expression of miR-637 was observed in tumor tissues and the low expression levels of miR-637 were correlated with poor survival of ESCC patients. In particular, plasma miR-637 could be used as a noninvasive biomarker for ESCC patients.

**Conclusions:**

These results implicate the potential application of miR-637 for diagnosis and prognosis of esophageal cancer.

**Supplementary Information:**

The online version contains supplementary material available at 10.1186/s40364-022-00424-x.

## Introduction

Esophageal carcinoma ranks the seventh most common cancer and the sixth most deadly cancer worldwide, according to global cancer statistics in 2018 [1]. There are two main histological types of esophageal cancer, esophageal adenocarcinoma and esophageal squamous cell carcinoma (ESCC) [2, 3]. ESCC accounts for more than 90% of all esophageal cancer cases in China, which are often diagnosed at an advanced stage with a poor prognosis [3–5]. Despite intensive research for newly developed treatment strategies including novel immunotherapy, the improvement in survival of patients with esophageal cancer remains unfavorable [6]. Therefore, there is an urgent need to explore novel prognostic biomarkers and therapeutic targets for esophageal cancer patients.

MicroRNAs (miRNAs), a family of short non-coding RNAs (~ 22 nucleotides), can modulate gene expression via specifically binding to the 3′-untranslated region (3′-UTR) of target mRNA, thereby inducing mRNA degradation [7–9]. Aberrant expression of miRNAs has been associated with various mechanisms of cancer progression, including oncogenesis, metastasis, drug resistance and immune escape [10]. Recently, an increasing number of studies have revealed that miRNAs play a key role in regulating cancer stem cells (CSCs) [11]. Depending on particular target genes, miRNAs function as either oncogenes or tumor suppressors, even in one type of cancer. For instance, down-regulation of miRNA-30e has been reported to increase cancer cell proliferation, invasion and tumor growth through targeting RPS6KB1 in esophageal cancer [12]. In contrast, miR-196b was significantly overexpressed and served as an oncogene to promote drug resistance in ESCC by targeting EPHA7 [13]. Furthermore, high expression of miR-17-5p and miR-4443 were closely correlated with tumor stages, suggesting a prognostic vale of the two miRNAs in ESCC [14]. Recently, emerging studies focused on the feasibility of blood miRNAs as noninvasive biomarkers for diagnosis and prognosis of cancers [15, 16].

WASH is a member of Wiskott-Aldrich syndrome protein (WASP) family and acts as nucleation-promoting factors for actin-related protein 2/3 complex (ARP2/3) to drive the generation of branched actin filaments [17]. WASH plays a pivotal role in distinct cellular biological processes, such as autophagy [18], mitosis [19], endosomal recycling [20] and phagosome maturation [21]. In our previous study, WASH was reported to enhance cancer stemness and associated with poor prognosis in ESCC patients [22]. However, the underlying mechanism of WASH expression remains largely unknown. Therefore, identification of miRNAs regulating the expression of WASH may offer new insights into prognostic biomarkers or therapeutic targets for ESCC.

In this study, we identified miR-637 as an important miRNA that directly targets WASH expression and inhibits cancer cell stemness via IL-8 signaling in vitro. We further demonstrated the therapeutic potential of miR-637 in xenograft tumor models of ESCC. Furthermore, loss of miR-637 in tumor tissue as well as peripheral blood was closely associated with poor prognosis of patients with ESCC. Overall, our findings provide significant evidence for miR-637 as a valuable diagnostic and prognostic biomarker for ESCC.

## Materials and methods

### Cell lines and cell culture

Human ESCC cell lines KYSE70 and KYSE450 were obtained from Cancer Hospital of Chinese Academy of Medical Sciences and Peking Union Medical College. All cancer cell lines were grown in RPMI 1640 medium (Sigma) supplemented with 10% heat-inactivated fetal bovine serum (FBS) (HyClone), 100 units/mL penicillin and 100 μg/mL streptomycin. Cells were cultured at 37 °C in a humidified incubator with 5% CO_2_.

### miRNA transfections

The mimic and inhibitor of miR-637, as well as the corresponding negative control (NC), were purchased from GenePharma (Shanghai, China). Cells were transfected with miR-637 mimic (100 nM) or inhibitor (100 nM) by using Lipofectamine 3000 transfection reagent (Invitrogen) according to the manufacturer’s instruction.

### RNA extraction and quantitative real-time PCR (qRT-PCR)

Total RNA was extracted from cancer cell lines, tumor tissue and plasma respectively, using RNAiso Plus (Takara Bio) according to the manufacturer’s instructions. The isolated total RNA was quantified by a NanoDrop spectrophotometer (Thermo Fisher Scientific). To quantify mRNA expression, total RNA was used to generate cDNA using PrimeScript RT reagent Kit with gDNA Eraser (Takara Bio). To measure the expression level of miR-637, total RNA was transcribed to cDNA using Mir-X miRNA First-Strand Synthesis Kit (Takara Bio). qRT-PCR was carried out using specific primers and SYBR Green Master Mix (Roche) on a Stratagene Mx3005P qPCR System (Agilent Technologies). The primer sequences are listed in Supplementary Table S1.

### Western blotting

Cells were lysed with RIPA buffer (Solarbio, China) containing protease inhibitor cocktail (Sigma-Aldrich). Cell lysates were sonicated on ice, resolved on SDS-PAGE and transferred to a nitrocellulose membrane. After blocking with 5% non-fat milk, the membranes were probed with primary antibodies for the detection of WASH (ab157592, Abcam) and β-actin (3700S, Cell Signaling Technology) followed by horseradish peroxidase-conjugated secondary antibodies (Cell Signaling Technology). Protein expression was detected using enhanced chemiluminescent HRP substrate (Thermo Fisher Scientific) and photographed with a Chemiluminescence Imaging System (Bio-Rad Laboratories).

### Dual-luciferase reporter assay

The 3′-UTR of WASH gene containing the predicted miR-637 binding site was synthesized by Sango Biotech (Shanghai, China) and cloned into the pmirGLO vector (Promega). The mutant construct with altered binding sites of miR-637 was generated by using the QuickChange Site-Directed Mutagenesis kit (Agilent Technologies). 293 T cells were transfected with the constructed pmirGLO vector (200 ng) and 50 nM miR-637 or NC mimic using Lipofectamine 3000 (Invitrogen). After 48 h of transfection, cells were collected and luciferase activity was measured using Dual-Luciferase Reporter Assay System (Promega). The firefly luciferase activity was normalized to Renilla luciferase activity.

### Enzyme-linked immunosorbent assay (ELISA)

Supernatant was collected from cell culture and plasma was isolated from peripheral blood, respectively. The IL-8 production was measured by using human IL-8 ELISA kit (BioLegend) according to the manufacturer’s instruction.

### Sphere formation assay

For enrichment of CSCs, cancer cells were seeded into ultra-low attachment 24-well culture plates (Corning) at a density of 20,000 cells/well and cultured in serum-free DMEM/ F12 medium (Gibco), containing 20 ng/mL human recombinant epidermal growth factor (Sigma-Aldrich), 20 ng/mL human recombinant basic fibroblast growth factor (Sigma-Aldrich), 1:50 dilution of B27 (Gibco) and 5 μg/mL insulin (Sigma-Aldrich). After 5 ~ 7 days of culture, the cells formed CSCs-like aggregates and the number of tumor spheres was counted under microscope.

### Lentivirus production and transduction

The lentiviral plasmid overexpressing IL-8 (OE-IL-8) was purchased from GeneChem (Shanghai, China). To produce lentiviral particles, the plasmid was transfected into 293 T cells with pMD2.G and psPAX2 using Lipofectamine 3000. Forty eight hours after transfection, viral supernatant was harvested and then transduced into KYSE450 cells with 8 μg/ml polybrene. After culture for several days, stable IL-8 overexpressing cells were obtained upon culture in the presence of 1 μg/ml puromycin (Invitrogen). For stable overexpression of miR-637, the miRNA lentiviral stocks carrying miR-637 and GFP were purchased from Genechem (Shanghai, China) and transduced KYSE450 cells according to the manufacturer’s instructions. The successfully transduced cells were sorted for GFP-positive cells by Moflo-XDP cytometer (Beckman Coulter).

### Cell proliferation assay

Cell proliferation was assessed using a Cell Counting Kit (CCK-8; Dojindo) according to the manufacturer’s protocol.

### Wound healing assay

Cells were seeded in a 6-well plate and incubated for 24 h. The monolayer was scratched by a sterile pipette tip and cultured in serum-free medium for 0, 24 and 48 h. The area of wound closure was measured by microscopic photography with Image J software.

### Transwell migration assay

Transwell migration assays were performed using uncoated inserts (8-μm pore size; Corning). Cells were placed in serum-free media into the upper chamber. The lower well contained medium 10% FBS. After 24 h incubation, cells that had migrated through the pores were fixed, stained with crystal violet solution, photographed and counted.

### Tumor xenograft mouse model

Female 6–8 weeks old NTG mice (NOD-scid IL2Rγ^−/−^) were purchased from SPF Beijing Biotechnology (China) and housed in specific pathogen-free animal laboratory. NTG mice were subcutaneously injected with KYSE450 cells stably overexpressing miR-637 or NC (3 × 10^6^ cells /mouse). Tumor size and body weight were measured twice a week. Tumor volume was calculated with the following formula: volume = (length **×** width^2^)/2. After 5 weeks, tumor xenografts were collected from the mice for qRT-PCR assays. The animal experiments were approved by the Animal Care and Use Committee of Zhengzhou University Animal Facility (Approval number: ZZU-LAC20200807).

### Immunohistochemistry (IHC) analysis

Paraffin-embedded mouse tumor xenografts were used to IHC analysis. After deparaffinization, rehydration and antigen retrieval, the tissue slides were incubated with primary anti-human IL-8 (Proteintech, 27,095–1-AP) or anti-human Ki67 (Servicebio, GB121141) antibodies, respectively. Immunological reactions were detected by horseradish peroxidase-conjugated secondary antibodies and diaminobenzidine substrate (Dako). The slides were quantitatively evaluated according to staining percentage and intensity, ranged from 0 (no staining) to 12 (100% of cells with intense staining).

### Flow cytometry analysis

Cells were re-suspended in PBS containing 2% FBS and stained with PE-conjugated anti-human CD44 antibody (BioLegend) at 4 °C for 30 min. PE-conjugated mouse IgG1 isotype control (BioLegend) was used as the control. The expression of surface CD44 was analyzed by FACS Canto II cytometer (BD Biosciences).

### Human esophageal cancer tissue and blood samples

All clinical samples were obtained from the First Affiliated Hospital of Zhengzhou University, China. The tumor tissue samples and adjacent normal tissue samples were collected from ESCC patients who underwent surgical resection at the Department of Thoracic Surgery. The clinical information of the patients from which these tissue samples were derived is shown in Supplementary Table S2. Peripheral blood samples were collected from ESCC patients and healthy donors (HD). The clinical information of the patients from which these peripheral blood samples were derived is shown in Supplementary Table S3. This study was approved by the Institutional Ethics Committee of the First Affiliated Hospital of Zhengzhou University (Approval number: 2019-KY-255).

### Statistical analysis

Data were analyzed by paired or unpaired Student’s t-test. Correlation analysis was performed with Pearson’s test. miR-637 expression with overall survival was analyzed using Log-Rank test. All statistical analyses were performed by using GraphPad Prism 7 software. ROC curves were plotted by the R language packages pROC and glmnet. *p* < 0.05 was considered statistically significant. Data were presented as the mean ± standard deviation from 2 to 3 independent experiments.

## Results

### miR-637 is a direct upstream regulator of WASH

**Fig. 1:**
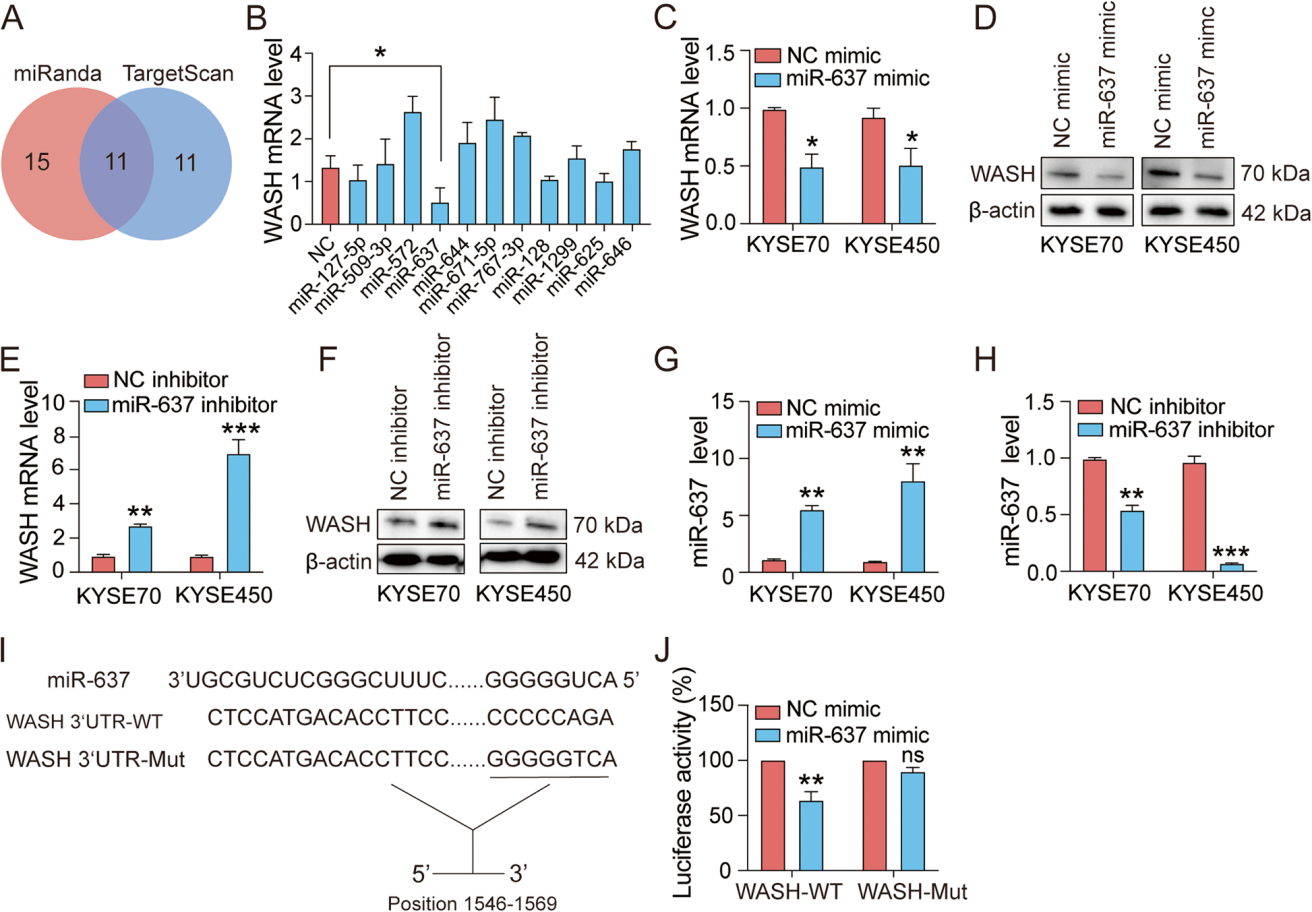
WASH is a direct target of miR-637 in ESCC cells. **A** The database miRanda and TargetScan were used to predict candidate miRNAs targeting WASH. **B** Relative expression of WASH was analyzed by qRT-PCR in KYSE70 cells transfected with the miRNA mimics for negative control (NC) or predicted candidates targeting WASH. **C**, **D** The down-regulation of WASH expression was determined in both KYSE70 and KYSE450 cells transfected with miR-637 mimic by qRT-PCR (**C**) and Western blotting (**D**), respectively. **E**,** F** The up-regulation of WASH expression was confirmed in both KYSE70 and KYSE450 cells transfected with miR-637 inhibitor by qRT-PCR (**E**) and Western blotting (**F**), respectively. **G**, **H** The relative levels of miR-637 were measured by qRT-PCR assays in KYSE70 cells and KYSE450 cells after transfection with mi-637 mimic (**G**) or miR-637 inhibitor (**H**), respectively. **I** Schematic representation of WASH 3′-UTR wild type (WT), and mutant (Mut) with altered residues in the core region of miR-637 binding site. **J** Luciferase activity in 293 T cells co-transfected with WASH WT-3′-UTR or Mut-3′-UTR constructs and miR-637 or NC mimic, respectively. Data are presented as mean ± standard deviation. ns, not significant; *p < 0.05, **p < 0.01, ***p < 0.001

We have previously reported that high WASH expression in ESCC tissue is significantly related to poor prognosis [22]. To dissect the molecular mechanism of WASH overexpression and the potential prognostic biomarker, we utilized TargetScan and miRanda to analyze miRNA-binding sites in the 3′-UTR of WASH. As a result, a total of eleven predicted miRNA candidates were screened out (Fig. 1A). To determine WASH-targeting miRNAs, specific miRNA mimics and a NC mimic were transfected into KYSE70 cells, respectively. Compared to the NC mimic, only miR-637 significantly suppressed WASH expression (Fig. 1B). We further confirmed that miR-637 mimic down-regulated WASH expression in two ESCC cell lines, at both mRNA and protein levels (Fig. 1C, D). In contrast, transfection of a miR-637 inhibitor resulted in a significant increase in the expression of WASH mRNA and protein (Fig. 1E, F). The successful regulation of miR-637 expression was shown in two ESCC cell lines upon transfection with miR-637 mimic or inhibitor, respectively (Fig. 1G, H). To validate whether WASH is a direct target of miR-637, we constructed the luciferase reporter plasmids containing wild-type or mutant 3′-UTR of WASH at the miR-637 binding site (Fig. 1I). The luciferase activity of 293 T cells transfected with the plasmid encoding wild-type 3′-UTR of WASH was remarkably reduced by miR-637 mimic, whereas the luciferase activity in 293 T cells transfected with the plasmid encoding mutant 3′-UTR of WASH was not affected (Fig. 1J). Collectively, these results clearly indicated that miR-637 down-regulates WASH expression through directly binding to the 3′-UTR of WASH.

### miR-637 inhibits IL-8 expression and CSCs properties in ESCC cells

**Fig. 2:**
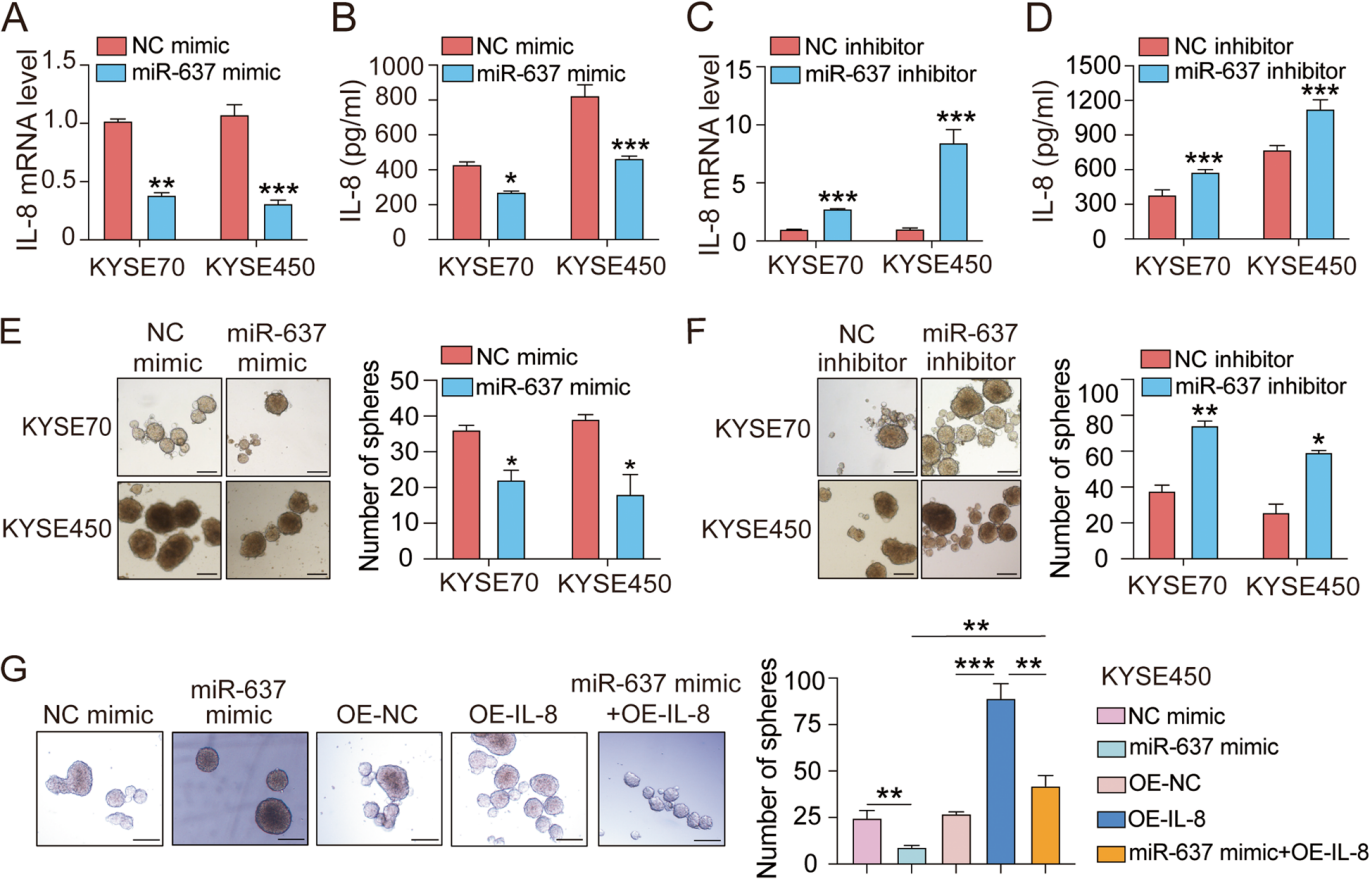
miR-637 suppresses stem-like characteristics in ESCC cells by inhibiting IL-8 expression. **A**, **B** qRT-PCR (**A**) and ELISA (**B**) assays showed the expression levels of IL-8 in KYSE70 cells and KYSE450 cells after treatment with negative control (NC) mimic or miR-637 mimic. **C**, **D** qRT-PCR (**C**) and ELISA (**D**) assays showed the expression levels of IL-8 in KYSE70 cells and KYSE450 cells after treatment with NC inhibitor or miR-637 inhibitor. **E**, **F** The number of spheres derived from KYSE70 cells and KYSE450 cells after treatment with treated with miR-637 mimic (**E**) or miR-637 inhibitor (**F**). **G** The number of spheres derived from IL-8 overexpressing (OE-IL-8) KYSE450 cells after treatment with miR-637 mimic. Scale bar, 200 μm. Data are presented as mean ± standard deviation. *p < 0.05, **p < 0.01, ***p < 0.001

Our previous data showed that WASH promoted the cancer stemness properties of ESCC through induction of IL-8 [22]. To identify the downstream effector of miR-637, the expression levels of IL-8 were analyzed by qRT-PCR and ELISA assays, respectively. The expression of IL-8 was found to be noticeably decreased after transfection of miR-637 mimic in two tested ESCC cell lines (KYSE70 and KYSE450), at transcription level (Fig. 2A) and protein level (Fig. 2B). Furthermore, treatment with miR-637 inhibitor consistently induced the increase of IL-8 production in KYSE70 and KYSE450 cells, at transcription level (Fig. 2C) and protein level (Fig. 2D). Next, the impact of miR-637 expression on the cancer stemness of ESCC cell lines was assessed by sphere formation assay in vitro. After incubation for 5–7 days, both KYSE70 cells and KYSE450 cells transfected with miR-637 mimic exhibited an inhibitory effect on tumor sphere formation (Fig. 2E). In contrast, transfection of miR-637 inhibitor resulted in a significant increase of the sphere formation capacity in the two cell lines (Fig. 2F). Consistently, the expression levels of stemness-related genes, including SOX4, SOX9, Nanog, CD44 and ABCG2, were also affected by miR-637 mimic (Supplementary Fig. S1A) or miR-637 inhibitor (Supplementary Fig. S1B), respectively. To confirm the role of IL-8 in miR-637-regulated tumor sphere formation, we generated IL-8 overexpressing KYSE450 cells (Supplementary Fig. S1C). As expected, ectopic expression of IL-8 promoted the formation of tumor spheres (Fig. 2G) and increased the expression of multiple stemness markers in KYSE450 cells (Fig. Supplementary S1D). Furthermore, IL-8 overexpression was able to rescue the miR-637-induced inhibition of tumor sphere formation (Fig. 2G) and stemness-related gene expression (Supplementary Fig. S1D). Similarly, addition of exogenous IL-8 also enhanced the CSCs properties and partially reversed the effect of miR-637 mimic in both KYSE70 cells (Supplementary Fig. S2A, B) and KYSE450 cells (Supplementary Fig. S2C, D). These data strongly indicated that miR-637 possesses a tumor suppressive role in ESCC through inhibition of IL-8 and cancer stemness.

### miR-637 suppresses cancer stemness of KYSE70 cells by a WASH-dependent mechanism

**Fig. 3:**
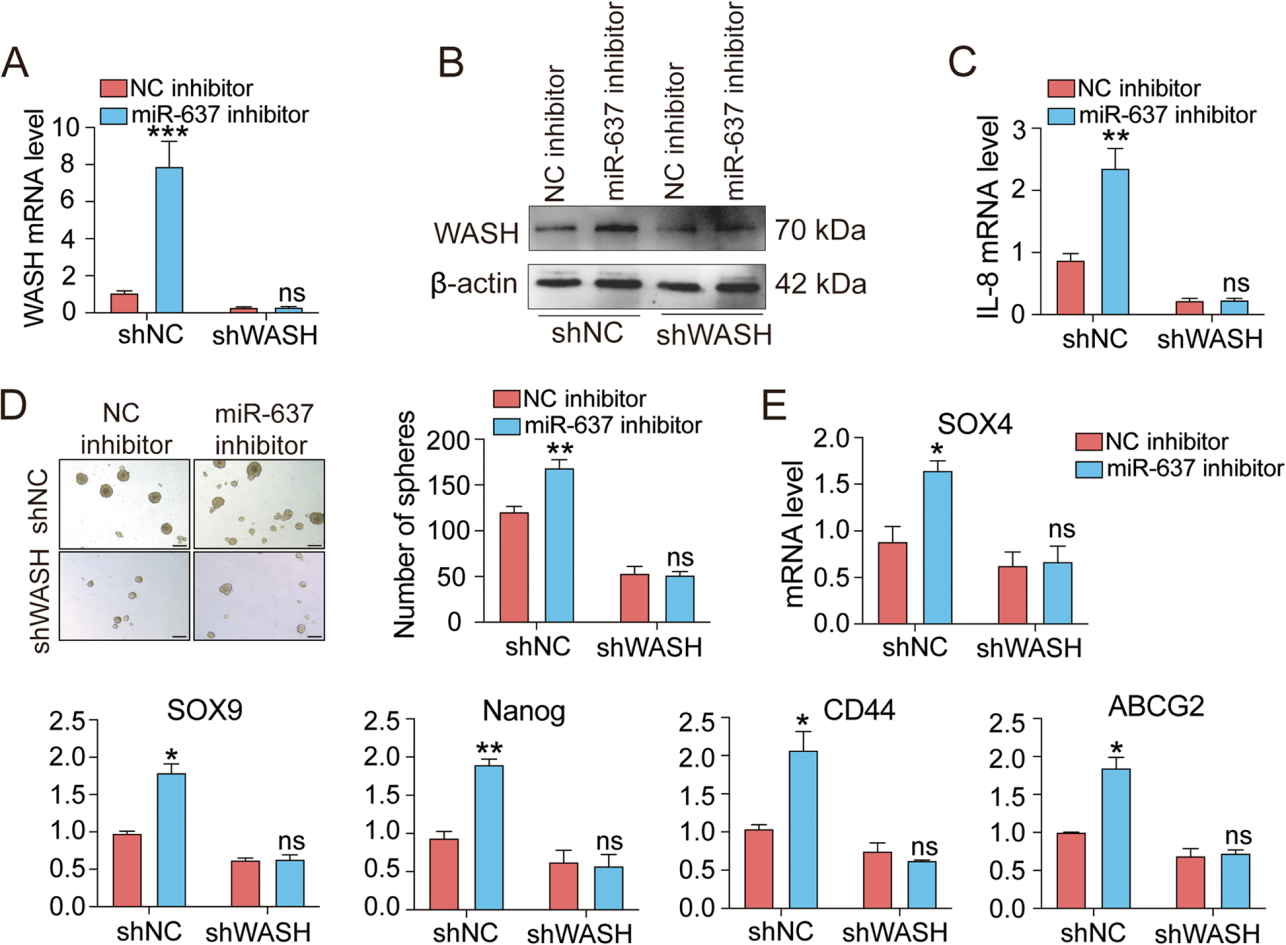
miR-637 inhibits IL-8 expression and cancer stemness in a WASH-dependent manner. KYSE70 cells stably transduced with negative control shRNA (shNC) or WASH shRNA (shWASH) were treated with NC inhibitor or miR-637 inhibitor. **A**, **B** qRT-PCR (**A**) or Western blotting (**B**) assays showed the failure of miR-637 inhibitor to induce WASH expression in KWSE70 cells with WASH knockdown. **C**, **D** qRT-PCR (**C**) or tumor sphere formation **(D)** assays showed the failure of miR-637 inhibitor to induce IL-8 expression and sphere formation capacity in KWSE70 cells with WASH knockdown, respectively. Scale bar, 200 μm. **E** The relative expression of stemness markers were measured by qRT-PCR. Data are presented as mean ± standard deviation. ns, not significant; *p < 0.05, **p < 0.01, ***p < 0.001

To further determine the role of WASH in miR-637-induced tumor suppression, a lentiviral-mediated approach was used to construct stable WASH knockdown ESCC cell line [22]. The successful knockdown of WASH expression was detected in KYSE70 cells at the mRNA (Supplementary Fig. S3A) and protein levels (Supplementary Fig. S3B). Furthermore, WASH knockdown decreased the expression of IL-8 in KYSE70 cells (Supplementary Fig. S3C). As expected, transfection of miR-637 inhibitor resulted in WASH expression in control cells but not WASH-knockdown cells both at the mRNA level (Fig. 3A) and protein level (Fig. 3B). IL-8 is a downstream target of WASH and promotes the stemness of ESCC cells. Similarly, miR-637 inhibitor increased the expression of IL-8 in control cells but not WASH-knockdown cells (Fig. 3C), indicating that miR-637 regulates IL-8 expression by targeting WASH. Finally, we analyzed miR-637 inhibitor-induced tumor sphere formation and stem cell markers after modulation of WASH expression. Again, transfection with miR-637 inhibitor failed to increase the number of tumor spheres and up-regulate the expression of stemness-related genes (SOX4, SOX9, Nanog, CD44 and ABCG2) after stable knockdown of WASH in KYSE70 cells (Fig. 3D, E). These results demonstrate that down-regulation of miR-637 facilitates the maintenance of cancer stemness in ESCC cells likely through WASH/IL-8 pathway.

### Stable overexpression of miR-637 suppresses ESCC growth in vivo

To evaluate the preclinical impact of miR-637 in ESCC, we established stably miR-637-overexpressing KYSE450 cells by using lentiviral expression system. Consistently, stable overexpression of miR-637 dramatically reduced the expression of WASH, IL-8 and stemness genes, as well as the number of spheres formed by KYSE450 cells (Supplementary Fig. S4A-G). In addition, miR-637 overexpression slightly suppressed cell proliferation, but had no effect on cell migration (Supplementary Fig. S4H-J).

**Fig. 4:**
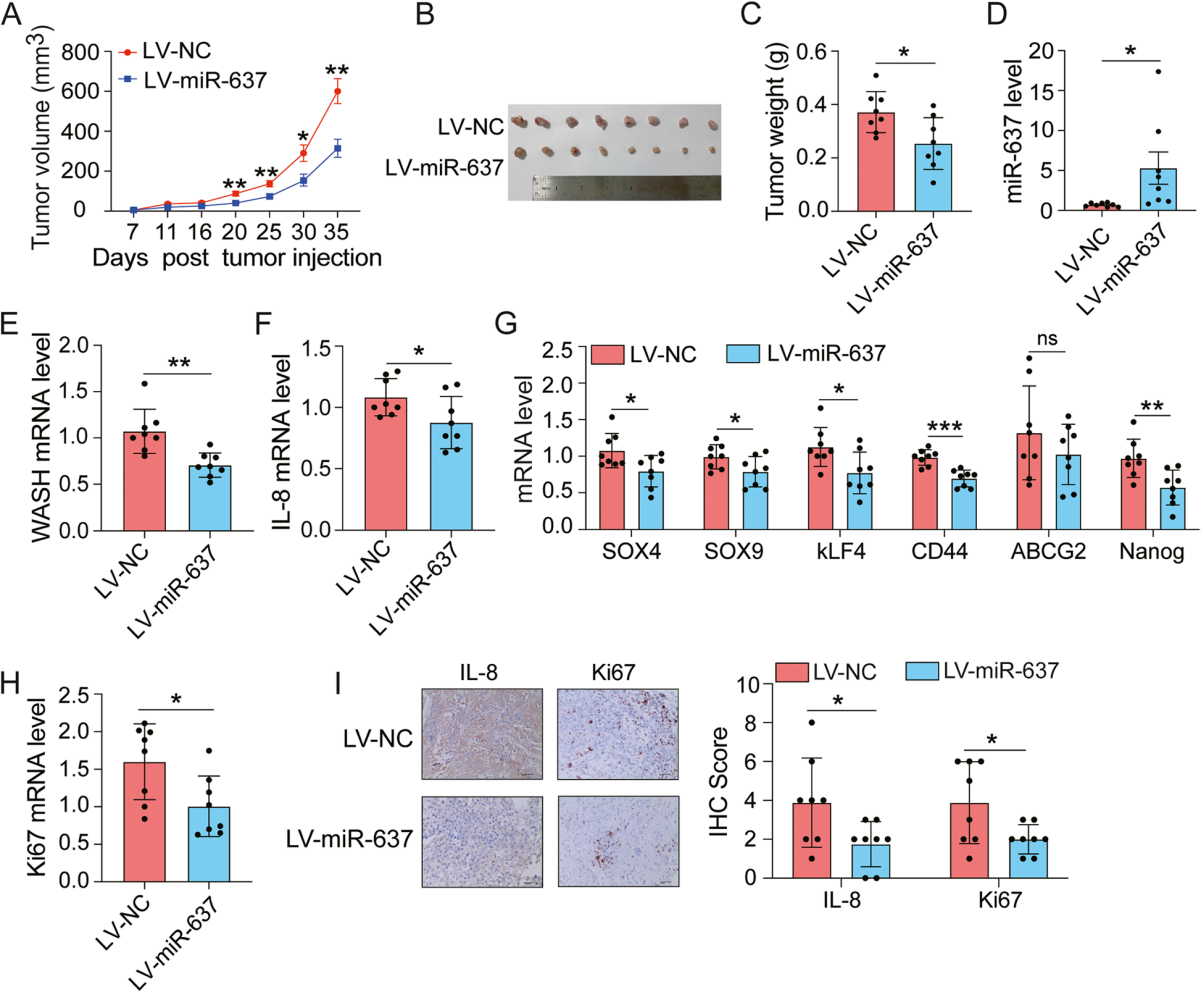
Ectopic expression of miR-637 reduces the growth of ESCC xenografts in mice. A total of 3 × 10^6^ KYSE450 cells stably transduced with lentivirus (LV) expressing miR-637 or negative control (NC) were implanted subcutaneously in immunodeficient NTG mice (*n* = 8 per group). **A** The volumes of transplanted tumors were measured at the indicated time points and the tumor growth curves were plotted. Data are presented as mean ± standard error of mean. **B** Images of all xenograft tumors excised at day 35 after tumor injection**. C** All tumor weights were measured. **D-H** The relative expression of miR-637 (**D**), WASH (**E**), IL-8 (**F**), stemness-related genes (**G**) and Ki67 (**H**) were examined by qRT-PCR, respectively. **I** IHC analysis of IL-8 and Ki67 expression in tumors. Scale bar, 40 μm. Data are presented as mean ± standard deviation. ns, not significant; *p < 0.05, **p < 0.01, ***p < 0.001

We examined the anti-tumor activity of miR-637 in a xenograft model. KYSE450 cells stably overexpressing miR-637 or control miRNA were subcutaneously injected into the flanks of NTG mice. Over a period of 5 weeks, ectopic expression of miR-637 leaded to an obvious attenuation in tumor growth curve (Fig. 4A), tumor size (Fig. 4B) and tumor weight (Fig. 4C) compared to control miRNA. Next, we detected the expression of miR-637 in KYSE450 xenografts by qRT-PCR (Fig. 4D). Consistent with our in vitro findings, miR-637 significantly inhibited the expression levels of WASH (Fig. 4E) and IL-8 (Fig. 4F) in KYSE450 xenografts. Similarly, the decreased levels of SOX4, SOX9, Nanog，CD44, KLF4 and ABCG2 were observed from the harvested KYSE450 tumors related to miR-637 overexpression, indicating the inhibition of cancer cell stemness by miR-637 (Fig. 4G). In addition, the expression of Ki67, a cell proliferation marker, decreased noticeably in miR-637 overexpressing KYSE450 xenografts (Fig. 4H). Furthermore, IHC analysis of tumor xenografts revealed that overexpression of miR-637 resulted in a significant reduction of IL-8 and Ki67 in protein level (Fig. 4I). Taken together, our data suggested the therapeutic potential of miR-637 in tumor bearing mice.

### miR-637 is down-regulated in tumor samples from ESCC patients

**Fig. 5:**
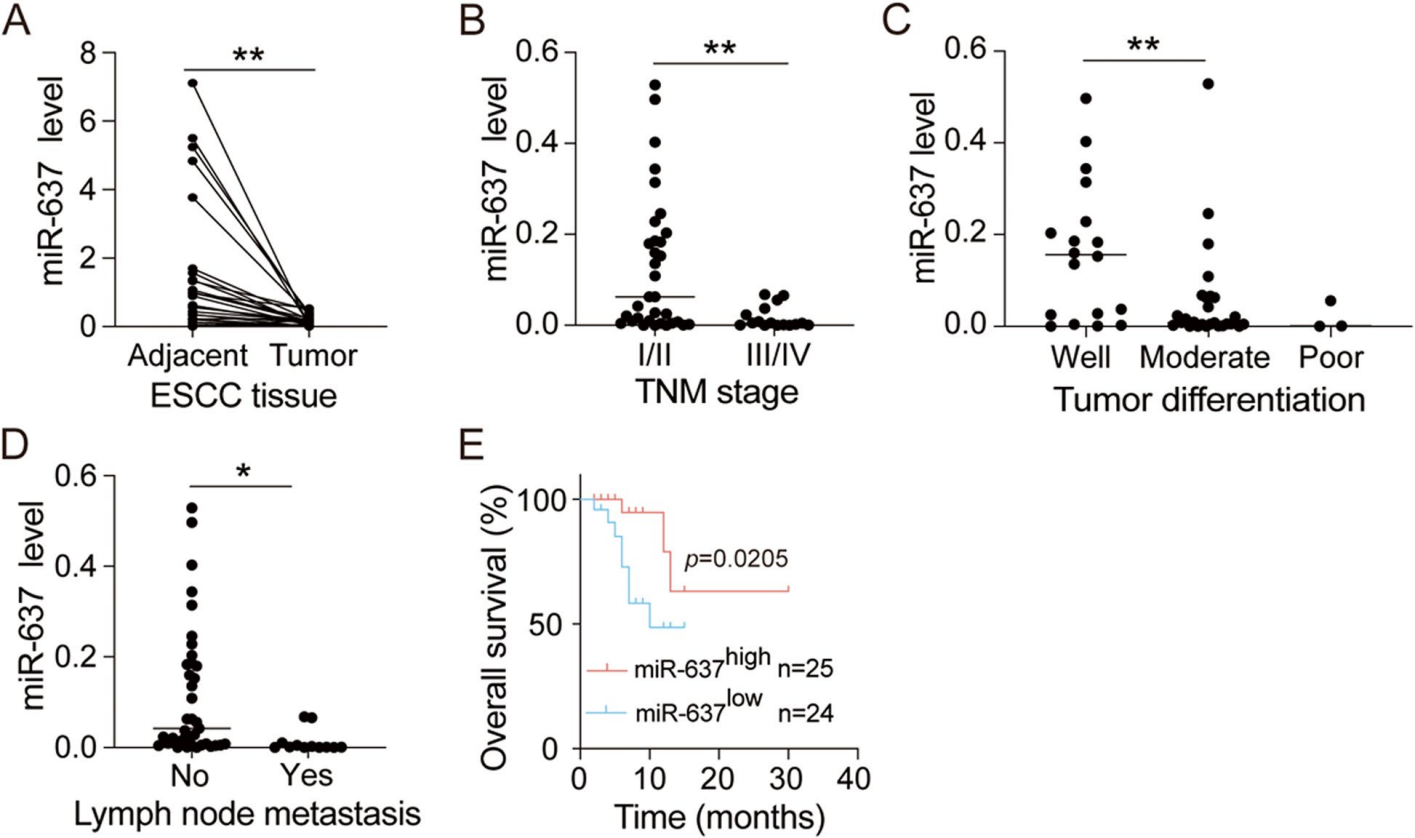
Low miR-637 expression in tumor tissues is associated with poor pathological features and overall survival of ESCC patients. **A** The relative expression of miR-637 was examined by qRT-PCR from paired tumor and adjacent normal tissues of 49 ESCC patients. **B-D** Decreased expression levels of miR-637 in tumor tissues correlate with advanced TNM stage (**B**), poor differentiation (**C**), and lymph node metastasis (**D**), respectively. **E** Kaplan-Meier survival analysis for overall survival was performed, comparing the relatively low and high miR-637 expression groups of ESCC patients. Data are presented as mean ± standard deviation. *p < 0.05, **p < 0.01

To determine the expression levels of miR-637 in human ESCC tissues, we performed qRT-PCR assay in 49 paired ESCC tumor and adjacent normal tissues. The expression level of miR-637 was significantly down-regulated in the tumor samples compared with adjacent normal tissues, revealing a possible tumor suppressive role of miR-637 in ESCC (Fig. 5A). Furthermore, the connection between miR-637 expression and pathological characteristics of ESCC was evaluated in 49 patients with ESCC. Low miR-637 expression was positively associated with late tumor-node-metastasis (TNM) stage (Fig. 5B), poor differentiation status (Fig. 5C) and lymph node metastasis (Fig. 5D). In Kaplan–Meier survival analysis, low miR-637 expression was positively correlated with poor overall survival of ESCC patients (Fig. 5E). Collectively, these data indicated that miR-637 was markedly down-regulated in tumor tissues and associated with poor prognosis of ESCC patients.

### miR-637 is a potential noninvasive biomarker for ESCC patients

**Fig. 6:**
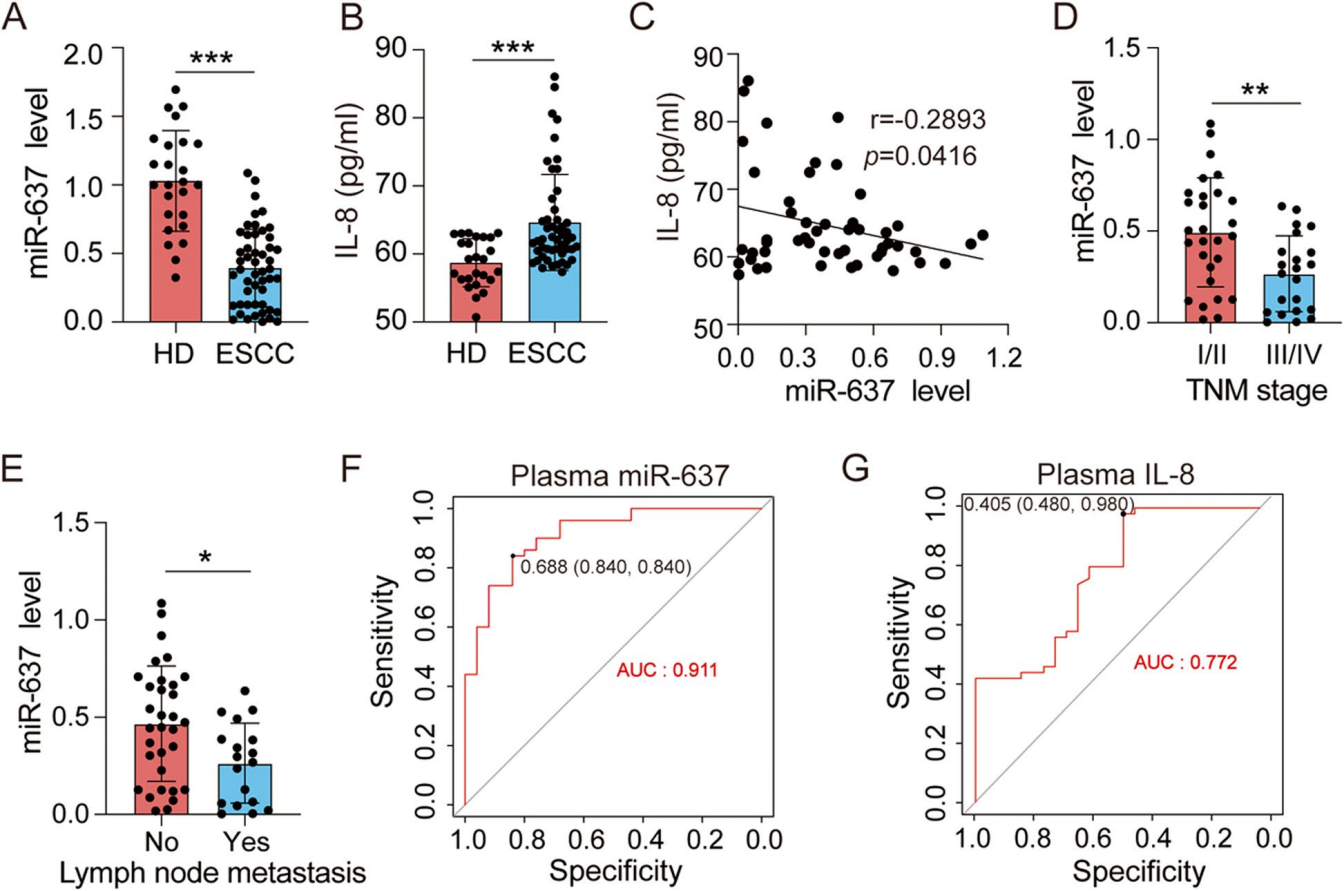
Plasma miR-637 is a useful biomarker for the diagnosis and prognosis of ESCC patients. **A**, **B** The expression levels of miR-637 and IL-8 in the plasma of healthy donors (HD) and ESCC patients were examined by qRT-PCR (**A**) and ELISA (**B**), respectively. **C** The relationship between miR-637 and IL-8 expression in the plasma of ESCC patients was evaluated via Pearson correlation analysis. **D**, **E** Low expression levels of plasma miR-637 correlate with advanced TNM stage (**D**) and lymph node metastasis (**E**). **F**, **G** The prediction performance of plasma miR-637 (**F**) or plasma IL-8 (**G**) in a cohort of ESCC patients were determined by ROC analysis. Data are presented as mean ± standard deviation. ns, not significant; *p < 0.05, **p < 0.01, ***p < 0.001

To explore the potential of miR-637 as a noninvasive biomarker for ESCC patients, the plasma samples from 50 ESCC patients and 25 healthy donors were used for qRT-PCR assays. We found that miR-637 expression was significantly decreased in the plasma from ESCC patients compared to that from healthy donors (Fig. 6A). Conversely, the levels of plasma IL-8 from ESCC patients were significantly higher than those from healthy donors (Fig. 6B). Pearson’s correlation analysis further confirmed that miR-637 and IL-8 expression were inversely correlated in ESCC plasma samples (Fig. 6C). Next, we analyzed the pathological relevance of plasma miR-637 and IL-8 in ESCC patients. Low levels of plasma miR-637 was positively correlated with advanced TNM stage (Fig. 6D) and lymph node metastasis (Fig. 6E). However, no connection between plasma IL-8 and TNM stage (Supplementary Fig. S5A) or lymph node metastasis (Supplementary Fig. S5B) was observed.

To evaluate the diagnostic potential of plasma miR-637 and IL-8 levels, we calculated the area under the curve (AUC) using a receiver operating characteristic (ROC) curve. The AUC of miR-637 and IL-8 was 0.911 and 0.772, respectively (Fig. 6F, G), indicating that plasma miR-637 is a better diagnostic biomarker for ESCC patients. In summary, loss of plasma miR-637 may serve as a useful noninvasive marker for diagnosis and prognosis of ESCC patients.

## Discussion

In recent years, the discovery of miRNAs has deepened our understanding of human cancers. Numerous studies have demonstrated the importance of miRNAs in cancer development and clinical application. In this present study, we demonstrated that miR-637 as a direct regulator of WASH promoted IL-8 production and cancer stemness properties of ESCC cells in vitro and in vivo. More importantly, miR-637 expression was down-regulated in ESCC tumor tissues and exhibited a negative correlation with patient survival. Plasma miR-637 was also lower in ESCC patients than healthy donors, indicating a potential novel biomarker of ESCC.

The members of the WASP family are primarily involved in actin polymerization and cytoskeleton reorganization, which is crucial for cancer development and metastasis [23]. Indeed, the WASP family proteins have been reported to be associated with adhesion, migration, invasion and colonization of malignant cells [24]. For instance, inhibition of N-WASP has been shown to reduce cancer stemness [25]. Data from our group also supports the participation of WASH in maintenance of cancer stemness [22]. However, there is little information about the regulation of WASH expression. Here, we identified that miR-637 could directly target the 3′-UTR of WASH and suppress its expression.

Previously, miR-637 has been reported to inhibit tumor progression in several types of cancers though various mechanisms [26–28]. In glioma, miR-637 represses tumor cell proliferation and migration by targeting Akt1 [26]. In addition, miR-637 retards glioblastoma progression via the ZEB2/WNT/beta-catenin cascades [27]. Moreover, miR-637 has been observed to inhibit tumor cell proliferation and invasion by targeting AKT1 in liver cancer [28]. Other studies also find the role of miR-637 in suppressing tumorigenesis and drug resistance [29, 30]. In line with these studies, we observed that inhibition of miR-637 greatly promoted IL-8 production and cancer stemness in a WASH-dependent manner, while overexpression of miR-637 showed an inverse effect both in vitro and in vivo. Interestingly, miR-637 slightly inhibited the cell proliferation but not migration of ESCC cell line KYSE450. Although IL-8 was reported to play multiple roles in tumor progression, recent studies demonstrated that IL-8 may promote CSCs-like properties [31, 32]. Here our work demonstrates IL-8 as a linker between miR-637 and cancer stemness properties, suggesting that miR-637 may inhibit cancer cell stemness through suppression of IL-8 in ESCC. These results indicate that miR-637 might serve as a negative regulator of cancer progression through inactivating several oncogenic pathways.

The present findings showed that miR-637 was markedly down-regulated in ESCC tissues and significantly correlated with TNM stage, tumor differentiation, lymph node metastasis and poor overall survival. In consistent with our study, miR-637 was reported significantly decreased in glioma tissues and positively associated with the prognosis of patients [26]. Recently, another study demonstrated that low expression of miR-637 was highly correlated with poor prognosis in patients with advanced breast cancer [33]. These findings support reduced expression of miR-637 as a novel biomarker for predication of advanced tumor features in ESCC patients.

Many studies have demonstrated that miRNAs circulate in the blood and can be easily detected by qRT-PCR [34]. Therefore, blood miRNAs may reflect the condition of tumor tissue and serve as a convenient noninvasive biomarker for cancer diagnosis and prognosis [35, 36]. Recently, microRNA array-based approaches have been widely used to explore circulating miRNAs as a biomarker for many types of cancers including ESCC [37–39]. In our study, miR-637 was significantly decreased in the plasma of ESCC patients compared to that in the plasma of healthy donors. Furthermore, the levels of plasma miR-637 was also found to be correlated with TNM stage and lymph node metastasis. This result suggests that miR-637 may be associated with tumor progression. Of note, we did not observe obvious correlations between plasma IL-8 and pathological prognostic parameters in ESCC. In addition to cancer cells, many other cell types including macrophages, lymphocytes and fibroblasts have also been shown to secrete IL-8 in tumor microenvironment [40]. As a diagnostic marker of ESCC, plasma miR-637 thus showed a better sensitivity compared to plasma IL-8. However, the clinical significance of plasma miR-637 should be interpreted cautiously since the sample size is relatively small in this study. To verify the diagnostic ability of plasma miR-637, further investigations need to be conducted on a larger number of patient samples and healthy donors.

## Conclusions

In summary, we found that miR-637 plays an important role as a tumor suppressor in ESCC cancer stemness through targeting WASH/IL-8 pathway. Additionally, decreased miR-637 expression is associated with poor prognosis. Thus, miR-637 may be a potential prognostic marker and therapeutic target for esophageal carcinoma.

## Supplementary Information

**Additional file 1: Supplementary Table S1.** Primer Sequences for qRT-PCR assays.

**Additional file 2: Supplementary Table S2.** Clinicopathological features of ESCC patients for tissue miR-637 analysis.

**Additional file 3: Supplementary Table S3.** Clinicopathological features of ESCC patients for plasma miR-637 analysis.

**Additional file 4: Supplementary Fig. S1.** Expression of stemness-related genes detected by qRT-PCR assays. A, B Regulation of SOX4, SOX9, Nanog, CD44 and ABCG2 expression in KYSE70 cells and KYSE450 cells after treatment with miR-637 mimic (A) or miR-637 inhibitor (B), respectively. C, D KYSE450 cells were stably transduced with lentivirus overexpressing (OE) negative control (NC) or IL-8. Identification of IL-8 overexpression (C) and stemness-related gene expression after treatment with miR-637 mimic (D). Data are presented as mean ± standard deviation. ns, not significant, *p < 0.05, **p < 0.01, ***p < 0.001.

**Additional file 5: Supplementary Fig. S2.** IL-8 is involved in miR-637-regulated cancer stemness of ESCC cell lines. KYSE70 cells (A, B) and KYSE450 cells (C, D) were transfected with negative control (NC) or miR-637 mimic in the presence of control or recombinant human IL-8 (100 ng/ml). Tumor sphere formation (A, C) and qRT-PCR (B, D) assays were performed respectively. Scale bar, 200 μm. Data are presented as mean ± standard. *p < 0.05, **p < 0.01, ***p < 0.001.

**Additional file 6: Supplementary Fig. S3.** Characteristics of KYSE70 cells with stable WASH knockdown. A, B Knockdown of WASH expression in KYSE70 cells through stable transduction with WASH shRNA (shWASH) or negative control shRNA (shNC) was validated by qRT-PCR (A) and Western blotting (B) assays, respectively. C The expression level of IL-8 was determined by qRT-PCR assay in KYSE70 cells with shRNA-mediated stable knockdown of WASH. Data are presented as mean ± standard deviation. ***p < 0.001.

**Additional file 7: Supplementary Fig. S4.** Characteristics of KYSE450 cells stably overexpressing miR-637. KYSE450 cells were stably transduced with lentivirus (LV) expressing miR-637 or negative control (NC). A-D The expression levels of miR-637 (A), WASH mRNA (B), IL-8 mRNA (C) and secreted IL-8 (D) were determined by qRT-PCR or ELISA assays. E-G The effects of stable miR-637 overexpression on cancer stemness were examined by tumor sphere formation (E), qPCR assay (F) and flow cytometry analysis (G). Scale bar, 200 μm. H-J CCK8 (H), wound healing (I) and Transwell migration (J) assays were used to detect cell proliferation and migration, respectively. Data are presented as mean ± standard deviation. ns, not significant, *p < 0.05, **p < 0.01, ***p < 0.001.

**Additional file 8: Supplementary Fig. S5.** Plasma IL-8 has no relationship with TNM stage and lymph node metastasis in ESCC patients. The expression levels of IL-8 in the plasma of ESCC patients were detected by ELISA assay and analyzed with TNM stage (A) and lymph node metastasis (B). ns, not significant.

## Data Availability

Authors declared that all and the other data supporting the findings of this study are available within the paper. The raw data that support the findings of this study are available from the corresponding author upon reasonable request.

## Abbreviations

AUC: Area under the curve
CSCs: Cancer stem cells
ESCC: Esophageal squamous cell carcinoma
FBS: Fetal bovine serum
HD: Healthy donors
IHC: Immunohistochemistry
IL-8: interleukin-8
LV: Lentivirus
miRNA: microRNA
NC: Negative control
OE: Overexpressing
ROC: Receiver operating characteristic
TNM: Tumor Node Metastasis
UTR: Untranslated region
WASH: Wiskott-Aldrich syndrome protein and SCAR Homolog
