# Supplementary material for: Loss of miR-637 promotes cancer cell stemness via WASH/IL-8 pathway and serves as a novel prognostic marker in esophageal squamous cell carcinoma
Source: Biomark Res. 2022 Nov 3;10:77. doi: 10.1186/s40364-022-00424-x (PMC9635169; doi:10.1186/s40364-022-00424-x)
Supplement: Supplementary file 1 [file 40364_2022_424_MOESM1_ESM.docx]

Supplementary Table 1. Primer Sequences for qRT-PCR assays

Gene	Primer Sequence
miR-637	Forward: 5'-ACTGGGGGCTTTCGGGCTCTGCGT-3'<br>Reverse: 5'-TCCAGTTTTTTTTTTTTTTTCGCA-3'
WASH	Forward: 5'-GGTGCCTGAGATCCATGTTCC-3'<br>Reverse: 5'-CCCATCTTGGTCTACCTTGA-3'
U6	Forward: 5'-CTCGCTTCGGCAGCACA-3'<br>Reverse: 5'-AACGCTTCACGAATTTGCGT-3'
IL-8	Forward: 5'-GAGAGTGATTGAGAGTGGACCAC-3'<br>Reverse: 5'-CACAACCCTCTGCACCCAGTTT-3'
GAPDH	Forward: 5'-CTGGGCTACACTGAGCACC-3'<br>Reverse: 5'-AAGTGGTCGTTGAGGGCAATG-3'
CD44	Forward: 5'-CTGCCGCTTTGCAGGTGTA-3'<br>Reverse: 5'-CATTGTGGGCAAGGTGCTATT-3'
KLF4	Forward: 5'-CCCACATGAAGCGACTTCCC-3'<br>Reverse: 5'-CAGGTCCAGGAGATCGTTGAA-3'
ABCG2	Forward: 5'-CAGGTGGAGGCAAATCTTCGT-3'<br>Reverse: 5'-ACCCTGTTAATCCGTTCGTTTT-3'
SOX4	Forward: 5'-AGCGACAAGATCCCTTTCATTC-3'<br>Reverse: 5'-CGTTGCCGGACTTCACCTT-3'
SOX9	Forward: 5'-AGCGAACGCACATCAAGAC-3'<br>Reverse: 5'-CTGTAGGCGATCTGTTGGGG-3'
Nanog	Forward: 5'-TCCCGAGAAAAGATTAGTCAGCA-3'<br>Reverse: 5'-AGTGGGGCACCTGTTTAACTT-3'
Ki67	Forward: 5'-GGGCCAATCCTGTCGCTTAAT-3'<br>Reverse: 5'-GTTATGCGCTTGCGAACCT-3'
