# Supplementary material for: Loss of miR-637 promotes cancer cell stemness via WASH/IL-8 pathway and serves as a novel prognostic marker in esophageal squamous cell carcinoma
Source: Biomark Res. 2022 Nov 3;10:77. doi: 10.1186/s40364-022-00424-x (PMC9635169; doi:10.1186/s40364-022-00424-x)
Supplement: Supplementary file 2 [file 40364_2022_424_MOESM2_ESM.docx]

Supplementary Table 2. Clinicopathological features of ESCC patients for tissue miR-637 analysis

Characteristics	Groups	No. of patients	Percentage (%)
Gender	Male	29	59
	Female	20	41
Age (years)	<60	18	37
	≥60	31	63
TNM stage	I/II	33	67
	III/IV	16	33
Tumor differentiation	Well	18	37
	Moderate	28	57
	Poor	3	6
Lymph node metastasis	No	37	76
	Yes	12	24
Follow-up status	Survival	38	78
	Death	11	22
	Loss to follow-up	0	0
