# Supplementary material for: Loss of miR-637 promotes cancer cell stemness via WASH/IL-8 pathway and serves as a novel prognostic marker in esophageal squamous cell carcinoma
Source: Biomark Res. 2022 Nov 3;10:77. doi: 10.1186/s40364-022-00424-x (PMC9635169; doi:10.1186/s40364-022-00424-x)
Supplement: Supplementary file 3 [file 40364_2022_424_MOESM3_ESM.docx]

Supplementary Table 3. Clinicopathological features of ESCC patients for plasma miR-637 analysis

Characteristics	Groups	No. of patients	Percentage (%)
Gender	Male	33	66
	Female	17	34
Age (years)	<60	20	40
	≥60	30	60
TNM stage	I/II	28	56
	III/IV	22	44
Lymph node metastasis	No	32	64
	Yes	18	36
