# Supplementary material for: Loss of miR-637 promotes cancer cell stemness via WASH/IL-8 pathway and serves as a novel prognostic marker in esophageal squamous cell carcinoma
Source: Biomark Res. 2022 Nov 3;10:77. doi: 10.1186/s40364-022-00424-x (PMC9635169; doi:10.1186/s40364-022-00424-x)
Supplement: Supplementary file 4 [file 40364_2022_424_MOESM4_ESM.docx]

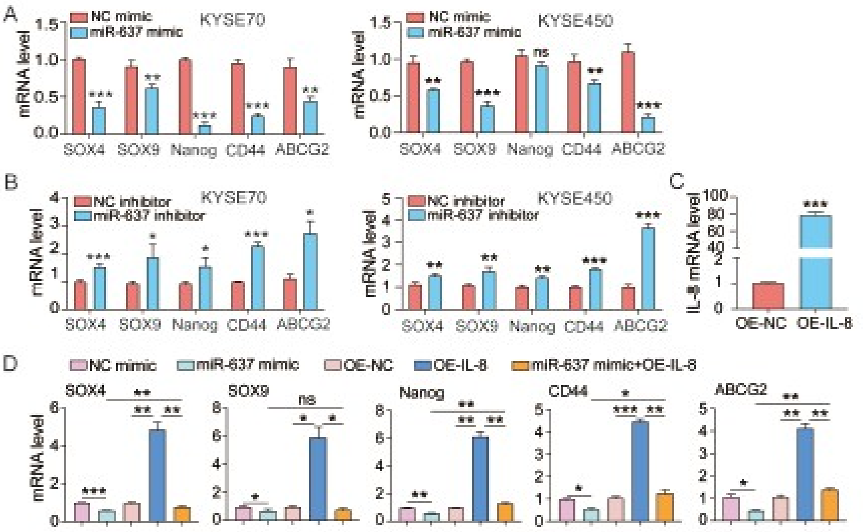

Supplementary Figure S1. Expression of stemness-related genes detected by qRT-PCR assays. **A, B** Regulation of SOX4, SOX9, Nanog, CD44 and ABCG2 expression in KYSE70 cells and KYSE450 cells after treatment with miR-637 mimic **(A)** or miR-637 inhibitor **(B)**, respectively. **C, D** KYSE450 cells were stably transduced with lentivirus overexpressing (OE) negative control (NC) or IL-8. Identification of IL-8 overexpression **(C)** and stemness-related gene expression after treatment with miR-637 mimic **(D)**. Data are presented as mean ± standard deviation. ns, not significant, *p<0.05, **p<0.01, ***p<0.001.
