# Supplementary material for: Loss of miR-637 promotes cancer cell stemness via WASH/IL-8 pathway and serves as a novel prognostic marker in esophageal squamous cell carcinoma
Source: Biomark Res. 2022 Nov 3;10:77. doi: 10.1186/s40364-022-00424-x (PMC9635169; doi:10.1186/s40364-022-00424-x)
Supplement: Supplementary file 5 [file 40364_2022_424_MOESM5_ESM.docx]

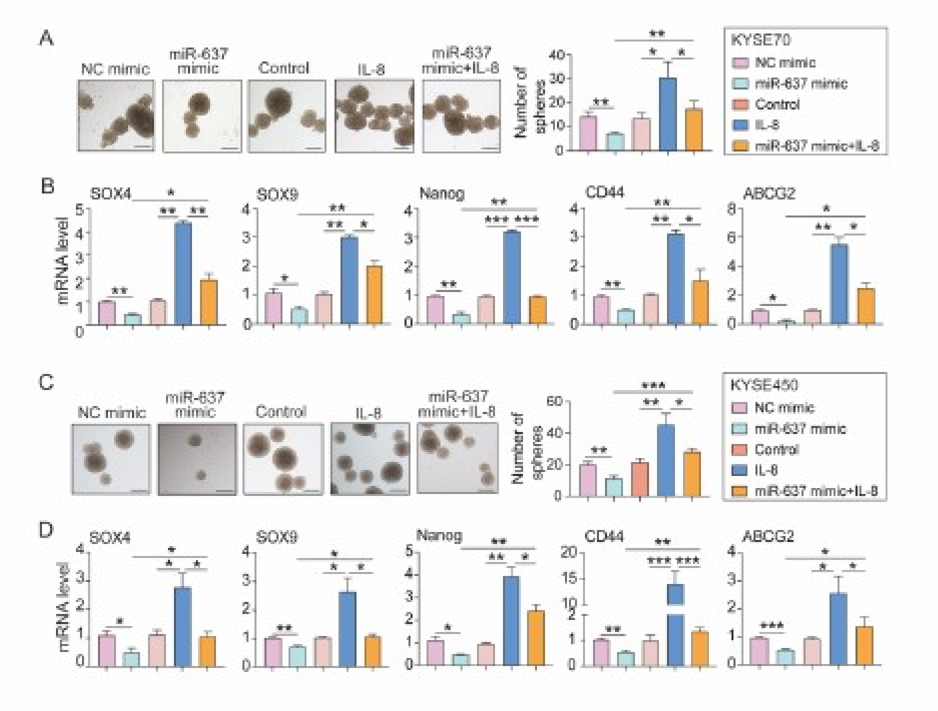

Supplementary Figure S2. IL-8 is involved in miR-637-regulated cancer stemness of ESCC cell lines. KYSE70 cells **(A, B)** and KYSE450 cells **(C, D)** were transfected with negative control (NC) or miR-637 mimic in the presence of control or recombinant human IL-8 (100 ng/ml). Tumor sphere formation **(A, C)** and qRT-PCR **(B, D)** assays were performed respectively. Scale bar, 200μm. Data are presented as mean ± standard. **p*<0.05, ***p*<0.01, ****p*<0.001.
