# Supplementary material for: Loss of miR-637 promotes cancer cell stemness via WASH/IL-8 pathway and serves as a novel prognostic marker in esophageal squamous cell carcinoma
Source: Biomark Res. 2022 Nov 3;10:77. doi: 10.1186/s40364-022-00424-x (PMC9635169; doi:10.1186/s40364-022-00424-x)
Supplement: Supplementary file 6 [file 40364_2022_424_MOESM6_ESM.docx]

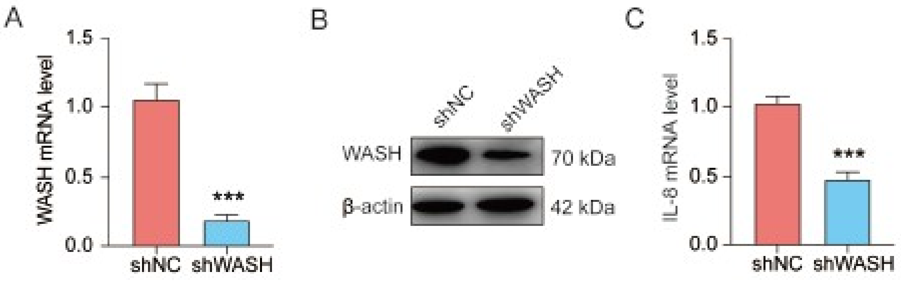

Supplementary Figure S3. Characteristics of KYSE70 cells with stable WASH knockdown. **A, B** Knockdown of WASH expression in KYSE70 cells through stable transduction with WASH shRNA (shWASH) or negative control shRNA (shNC) was validated by qRT-PCR **(A)** and Western blotting **(B)** assays, respectively. **C** The expression level of IL-8 was determined by qRT-PCR assay in KYSE70 cells with shRNA-mediated stable knockdown of WASH. Data are presented as mean ± standard deviation. ***p<0.001.
