# Supplementary material for: Loss of miR-637 promotes cancer cell stemness via WASH/IL-8 pathway and serves as a novel prognostic marker in esophageal squamous cell carcinoma
Source: Biomark Res. 2022 Nov 3;10:77. doi: 10.1186/s40364-022-00424-x (PMC9635169; doi:10.1186/s40364-022-00424-x)
Supplement: Supplementary file 7 [file 40364_2022_424_MOESM7_ESM.docx]

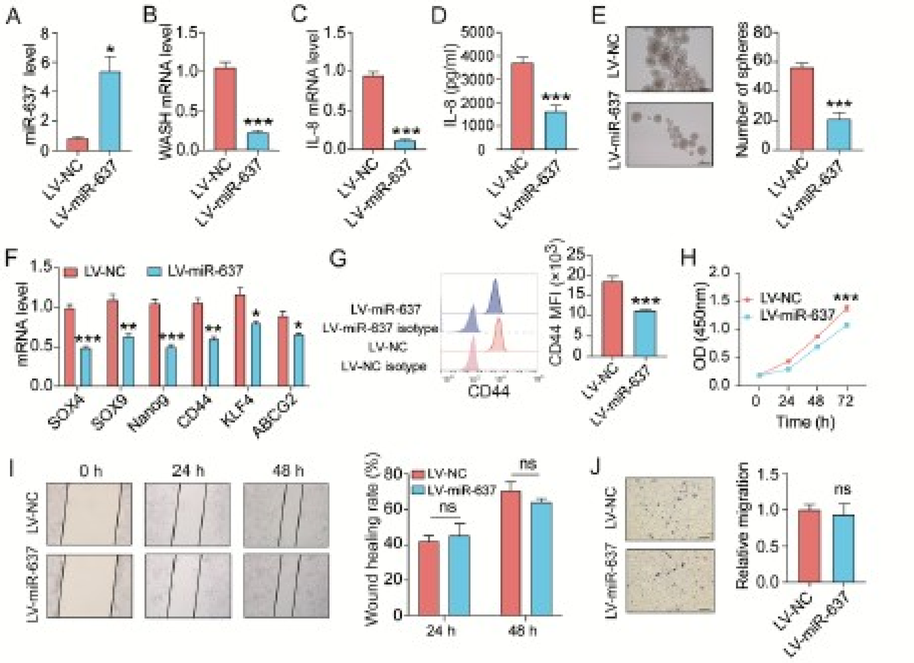

Supplementary Figure S4. Characteristics of KYSE450 cells stably overexpressing miR-637. KYSE450 cells were stably transduced with lentivirus (LV) expressing miR-637 or negative control (NC). **A-D** The expression levels of miR-637 **(A)**, WASH mRNA **(B)**, IL-8 mRNA **(C)** and secreted IL-8 **(D)** were determined by qRT-PCR or ELISA assays. **E-G** The effects of stable miR-637 overexpression on cancer stemness were examined by tumor sphere formation **(E)**, qPCR assay **(F)** and flow cytometry analysis **(G)**. Scale bar, 200 μm. **H-J** CCK8 **(H)**, wound healing **(I)** and Transwell migration **(J)** assays were used to detect cell proliferation and migration, respectively. Data are presented as mean ± standard deviation. ns, not significant, *p<0.05, **p<0.01, ***p<0.001.
