# Supplementary material for: Loss of miR-637 promotes cancer cell stemness via WASH/IL-8 pathway and serves as a novel prognostic marker in esophageal squamous cell carcinoma
Source: Biomark Res. 2022 Nov 3;10:77. doi: 10.1186/s40364-022-00424-x (PMC9635169; doi:10.1186/s40364-022-00424-x)
Supplement: Supplementary file 8 [file 40364_2022_424_MOESM8_ESM.docx]

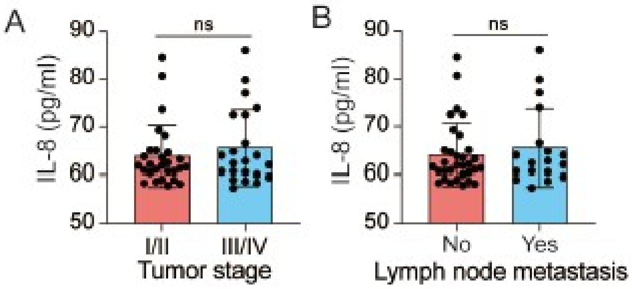


**Supplementary Figure S5.** Plasma IL-8 has no relationship with TNM stage and lymph node metastasis in ESCC patients. The expression levels of IL-8 in the plasma of ESCC patients were detected by ELISA assay and analyzed with TNM stage **(A)** and lymph node metastasis **(B)**. ns, not significant.
